# Nanocluster growth *via* “graft-onto”: effects on geometric structures and optical properties†
†Electronic supplementary information (ESI) available: Fig. S1–S14 and Tables S1–S3 for the EDS, ESI-MS, XPS, ICP, TGA, and stability and PL results of nanoclusters, and the structural comparison between nanoclusters. CCDC 1937755. For ESI and crystallographic data in CIF or other electronic format see DOI: 10.1039/c9sc05700e


**DOI:** 10.1039/c9sc05700e

**Published:** 2019-12-27

**Authors:** Xi Kang, Shan Jin, Lin Xiong, Xiao Wei, Manman Zhou, Chenwanli Qin, Yong Pei, Shuxin Wang, Manzhou Zhu

**Affiliations:** a Department of Chemistry and Centre for Atomic Engineering of Advanced Materials , Anhui Province Key Laboratory of Chemistry for Inorganic/Organic Hybrid Functionalized Materials , Anhui University , Hefei , Anhui 230601 , P. R. China . Email: ixing@ahu.edu.cn ; Email: zmz@ahu.edu.cn; b Institutes of Physical Science and Information Technology , Anhui University , Hefei , Anhui 230601 , P. R. China; c Key Laboratory of Structure and Functional Regulation of Hybrid Materials , Anhui University , Ministry of Education , Hefei , 230601 , P. R. China; d Department of Chemistry , Key Laboratory of Environmentally Friendly Chemistry and Applications of Ministry of Education , Xiangtan University , Xiangtan , Hunan 411105 , China

## Abstract

Atomically precise engineering on the nanocluster surface remains highly desirable for the fundamental understanding of how surface structures of a nanocluster contribute to its overall properties. In this paper, the concept of “graft-onto” has been exploited to facilitate nanocluster growth on surface structures. Specifically, the Ag_2_(DPPM)Cl_2_ complex is used for re-constructing the surface structure of **Pt_1_Ag_28_**(SR)_18_(PPh_3_)_4_ (**Pt_1_Ag_28_**, SR = 1-adamantanethiolate) and producing a size-growth nanocluster – **Pt_1_Ag_31_**(SR)_16_(DPPM)_3_Cl_3_ (**Pt_1_Ag_31_**). The grafting effect of Ag_2_(DPPM)Cl_2_ induces both direct changes on the surface structure (*e.g.*, size growth, structural transformation, and surface rotation) and indirect changes on the kernel structure (from a fcc configuration to an icosahedral configuration). Remarkable differences have been observed by comparing optical properties between **Pt_1_Ag_28_** and **Pt_1_Ag_31_**. Significantly, **Pt_1_Ag_31_** exhibits high photo-luminescent intensity with a quantum yield of 29.3%, which is six times that of the **Pt_1_Ag_28_**. Overall, this work presents a new approach (*i.e.*, graft-onto) for the precise dictation of nanocluster surface structures at the atomic level.

## Abstract 2

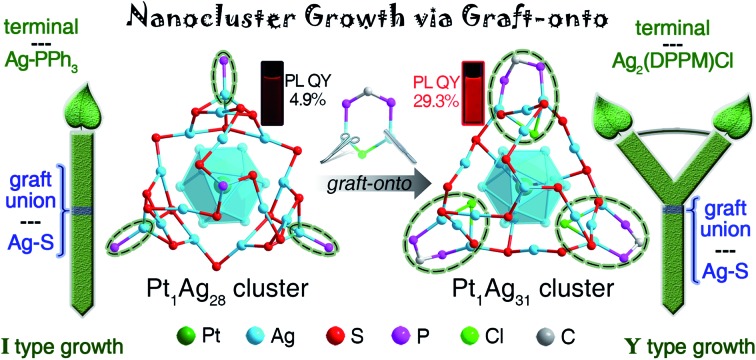
The concept of “graft-onto” has been exploited to facilitate nanocluster growth from **Pt_1_Ag_28_** to **Pt_1_Ag_31_**.

## 1 Introduction

It has long been nano-scientists' dream to control compositions and structures of nanomaterials at the atomic level. Through the continuous accumulation of synthetic experience and with the help of advanced analytical methods, researchers can now easily tailor the composition and the morphology of metal nanoparticles.^1^,^2^ However, it still remains extremely difficult to realize the atomic-level tailoring of specific sites on the nanoparticle surface (for example, adding or deleting one or two metal atoms at a designated position), which is the most relevant to the physical–chemical properties of such nanomaterials.

Nanoclusters (so-called ultrasmall nanoparticles) are an emerging class of promising nanomaterials owing to their atomically precise structures and intriguing properties.^3^,^4^ Because of the quantum size effect of nanoclusters, any perturbations on compositions/structures may induce tremendous variations on clusters' properties.^3^–^6^ Knowledge and understanding of these structure–property correlations are keys to the ultimate goal in the nanocluster science – arbitrarily dictating the properties *via* precisely tailoring the structures. Exploring effective approaches to exquisitely tailor the size, structure, and composition of atomically precise nanoclusters is a prerequisite to achieve this goal.

In recent years, ligand engineering has served as an efficient approach to convert structures of nanoclusters.^7^ In general, great structural transformation occurs when the peripheral ligands of nanoclusters are drastically substituted by the introduced ligands (for instance, from mono-icosahedral Au_11_(PPh_3_)_3_Cl_3_ to bi-icosahedral Au_25_(PPh_3_)_10_(SR)_5_Cl_2_,7*a* from Ag_44_(SR)_30_ with a hollow kernel to Ag_25_(SR)_18_ with a nonhollow kernel,7*b* and so on). However, it still remains challenging to tailor specific sites on the nanocluster surface without constructing the overall structure. A new approach for tailoring the nanocluster surface is highly desirable for the fundamental understanding of how surface structures in a nanocluster contribute to its overall properties.

By noting two facts about nanoclusters that (i) it is hard to substitute thiol ligands on the nanocluster surface by the introduced phosphorus ligands (because the metal–S covalent bond is more robust than the metal–P coordination bond) and (ii) several thiolated nanoclusters are terminally capped by metal–PPh_3_ units (such as **Pt_1_Ag_28_**(SR)_18_(PPh_3_)_4_, Ag_29_(SSR)_12_(PPh_3_)_4_, and Ag_33_(SR)_24_(PPh_3_)_4_ clusters with Ag–PPh_3_ terminals),^8^ we perceive a good opportunity to re-construct the nanocluster surface without largely affecting its whole structure – substituting these terminal metal–PPh_3_ units by bidentate phosphorus metal complexes. Such a substitution may not only fine-tune the nanocluster surface structure, but also shed light on structure–property correlations at the atomic level.

In the current work, we report a “graft-onto” strategy to facilitate a controllable size-growth of the nanocluster surface. Induced by the addition of the Ag_2_(DPPM)Cl_2_ complex (DPPM = bis-(diphenylphosphino)-methane), **Pt_1_Ag_28_**(SR)_18_(PPh_3_)_4_ (**Pt_1_Ag_28_**; SR = 1-adamantanethiolate) converts into a size-growth nanocluster – **Pt_1_Ag_31_**(SR)_16_(DPPM)_3_Cl_3_ (**Pt_1_Ag_31_**). Great changes (including size growth, structural transformation, and surface rotation) take place on the outermost shell of **Pt_1_Ag_28_**, owing to the direct grafting effect of the Ag_2_(DPPM)Cl_2_. The changes on the outermost shell further induce the transformation of the innermost Pt_1_Ag_12_ kernel from a fcc configuration in **Pt_1_Ag_28_** to an icosahedral configuration in **Pt_1_Ag_31_**. **Pt_1_Ag_28_** and **Pt_1_Ag_31_** nanoclusters reflect remarkable differences in both optical absorption and PL emission. Significantly, **Pt_1_Ag_31_** displays high photo-luminescence (PL) intensity with a quantum yield (QY) of 29.3%, which is six times that of the **Pt_1_Ag_28_** (PL QY = 4.9%).

## 2 Experimental methods

### Materials

All reagents were purchased from Acros Organics and Sigma-Aldrich and used without further purification: hexachloroplatinic(iv) acid (H_2_PtCl_6_·6H_2_O, 99%, metals basis), silver nitrate (AgNO_3_, 99% metals basis), adamantane-1-thiol (C_10_H_15_SH, HS-Adm, 95%), triphenylphosphine (PPh_3_, 95%), bis(diphenylphosphino)methane ((C_6_H_5_)_2_PCH_2_P(C_6_H_5_)_2_, DPPM, 98%), sodium borohydride (NaBH_4_, 99.9%), sodium chloride (NaCl, 99.5%), sodium hexafluoroantimonate (NaSbF_6_, 99%), rhodamine B (RB, for fluorescence), methylene chloride (CH_2_Cl_2_, HPLC, Aldrich), methanol (CH_3_OH, HPLC, Aldrich), ethyl acetate (CH_3_COOC_2_H_5_, HPLC, Aldrich), ethanol (CH_3_CH_2_OH, HPLC, Aldrich), ether (C_2_H_5_OC_2_H_5_, HPLC, Aldrich), and 2-methyltetrahydrofuran (C_4_H_7_O-2-CH_3_, HPLC, Aldrich).

### Synthesis of the [**Pt_1_Ag_28_**(S-Adm)_18_(PPh_3_)_4_]Cl_2_ nanocluster

For the nanocluster synthesis, AgNO_3_ (29 mg, 0.17 mmol) and H_2_PtCl_6_·6H_2_O (5 mg, 0.01 mmol) were dissolved in CH_3_OH (5 mL) and CH_3_COOC_2_H_5_ (35 mL). The solution was vigorously stirred (1200 rpm) with magnetic stirring for 15 min. Then, Adm-SH (0.1 g) and PPh_3_ (0.1 g) were added and the reaction was vigorously stirred (1200 rpm) for another 90 min. After this, NaBH_4_ aqueous solution (1 mL, 20 mg mL^–1^) was added quickly to the above mixture. The reaction was allowed to proceed for 36 h under a N_2_ atmosphere. After this, the aqueous layer was removed, and the mixture in the organic phase was rotavaporated under vacuum. Then approximately 30 × 3 mL of CH_3_CH_2_OH was used to wash the obtained nanoclusters. The precipitate was dissolved in CH_2_Cl_2_, which produced the [**Pt_1_Ag_28_**(S-Adm)_18_(PPh_3_)_4_]Cl_2_ nanocluster. The yield is 45% based on the Ag element (calculated from AgNO_3_) for the synthesis of [**Pt_1_Ag_28_**(S-Adm)_18_(PPh_3_)_4_]Cl_2_.

### Synthesis of the Ag_2_(DPPM)Cl_2_ complex

0.17 g of AgNO_3_ (1 mmol) was dissolved in 50 mL of CH_3_CH_2_OH, and NaCl aqueous solution (6 mL, 10 mg mL^–1^) was added quickly to the above mixture. The reaction was stirred for 1 minute. The white precipitate was collected and rotavaporated under vacuum, which produced the AgCl powder. Then, 0.07 g of AgCl (0.5 mmol) was dispersed in 20 mL of CH_2_Cl_2_, to which solution 0.19 g DPPM was added. The reaction was vigorously stirred (1200 rpm) with magnetic stirring for 30 minutes. After this, the solution was evaporated to dryness, which produced the Ag_2_(DPPM)Cl_2_ complex. The yield is about 95% based on the Ag element (calculated from AgCl) for the synthesis of Ag_2_(DPPM)Cl_2_.

### Synthesis of the [**Pt_1_Ag_31_**(S-Adm)_16_(DPPM)_3_Cl_3_]Cl_4_ nanocluster

For the nanocluster synthesis, 30 mg of [**Pt_1_Ag_28_**(S-Adm)_18_(PPh_3_)_4_]Cl_2_ was dissolved in 30 mL of CH_2_Cl_2_, to which 10 mg of Ag_2_(DPPM)Cl_2_ was added. The reaction was allowed to proceed for 10 minutes at room temperature. After this, the organic layer was separated from the precipitate and evaporated to dryness. Then, approximately 30 × 3 mL of CH_3_CH_2_OH was used to wash the obtained nanoclusters. The precipitate was dissolved in CH_2_Cl_2_, which produced the [**Pt_1_Ag_31_**(S-Adm)_16_(DPPM)_3_Cl_3_]Cl_4_ nanocluster. The yield is about 85% based on the Ag element (calculated from the **Pt_1_Ag_28_**) for the synthesis of [**Pt_1_Ag_31_**(S-Adm)_16_(DPPM)_3_Cl_3_]Cl_4_.

### Single-crystal growth of [**Pt_1_Ag_31_**(S-Adm)_16_(DPPM)_3_Cl_3_](SbF_6_)_4_

For accelerating the crystallization process and improving the quality of crystals, the counterion Cl^–^ in [**Pt_1_Ag_31_**(S-Adm)_16_(DPPM)_3_Cl_3_]Cl_4_ was replaced by SbF_6_^–^. The reaction equation is [**Pt_1_Ag_31_**(S-Adm)_16_(DPPM)_3_Cl_3_]Cl_4_ + 4 SbF_6_^–^ → [**Pt_1_Ag_31_**(S-Adm)_16_(DPPM)_3_Cl_3_](SbF_6_)_4_ + 4Cl^–^. Specifically, 20 mg of [**Pt_1_Ag_31_**(S-Adm)_16_(DPPM)_3_Cl_3_]Cl_4_ was dissolved in 20 mL of CH_2_Cl_2_. Then, 1 mL of NaSbF_6_–CH_3_CH_2_OH solution (5 mg mL^–1^) was added. After 3 minutes, the organic layer was separated from the precipitate and evaporated to dryness. The precipitate was dissolved in CH_2_Cl_2_, which produced the [**Pt_1_Ag_31_**(S-Adm)_16_(DPPM)_3_Cl_3_](SbF_6_)_4_ nanocluster. Nanoclusters were crystallized in a CH_2_Cl_2_/ether system with a vapor diffusion method. Specifically, 20 mg of clusters was dissolved in 5 mL of CH_2_Cl_2_, and the obtained solution was then vapor diffused using 50 mL of ether. After 3 days, dark red crystals of **Pt_1_Ag_31_** were collected and subjected to X-ray diffraction to determine the structure. The CCDC number of [**Pt_1_Ag_31_**(S-Adm)_16_(DPPM)_3_Cl_3_](SbF_6_)_4_ is ; 1937755. Notably, the optical absorption and PL emission properties of the **Pt_1_Ag_31_** nanocluster remain unchanged after the counter-ion replacement.

### Test of the temperature-photoluminescence (PL) intensity correlation

The nanocluster (0.1 mg) was dissolved in 5 mL of the CH_2_Cl_2_/C_4_H_7_O-2-CH_3_ (v/v = 1 : 1) mixture. Then, the solutions were cooled from 293 K to different temperatures and the PL spectra were measured.

### X-ray crystallography

The data collection for single crystal X-ray diffraction was carried out on a Bruker Smart APEX II CCD diffractometer under a nitrogen flow at 170 K, using graphite-monochromatized Mo Kα radiation (*λ* = 0.71073 Å). Data reductions and absorption corrections were performed using the SAINT and SADABS programs, respectively.9*a* The electron density was squeezed using PLATON, and detailed information can be found in Table S3.† The structure was solved by direct methods and refined with full-matrix least squares on *F*^2^ using the SHELXTL software package.9*b* All non-hydrogen atoms were refined anisotropically, and all the hydrogen atoms were set in geometrically calculated positions and refined isotropically using a riding model.

### Theoretical methods

Density functional theory (DFT) calculations were employed to optimize the geometric structures and calculated the Kohn–Sham orbitals of **Pt_1_Ag_28_** and **Pt_1_Ag_31_** nanoclusters using the Perdew–Burke–Ernzerhof (PBE) GGA functional.10*a* The triple-zeta polarized (TZP) basis set with inclusion of the scalar relativistic effect *via* a zeroth-order regular approximation (ZORA) implemented in the ADF package was adopted.10*b*

### Characterization

All UV-vis absorption spectra of the nanoclusters dissolved in CH_2_Cl_2_ were recorded using an Agilent 8453 diode array spectrometer, whose background correction was made using a CH_2_Cl_2_ blank.

Photo-luminescence (PL) spectra were measured on an FL-4500 spectrofluorometer with the same optical density (OD) of 0.05. In these experiments, the nanocluster solutions were prepared in CH_2_Cl_2_ at a concentration of less than 1 mg mL^–1^.

Absolute quantum yield (QY) was measured with dilute solutions of nanoclusters on a HORIBA FluoroMax-4P. For determining the QYs of clusters, the nanocluster solutions were prepared in CH_2_Cl_2_ with the same OD of 0.05. Besides, the PL comparison between the **Pt_1_Ag_31_**(S-Adm)_16_(DPPM)_3_Cl_3_ nanocluster and rhodamine B was performed, to further determine the PL QY of the **Pt_1_Ag_31_**(S-Adm)_16_(DPPM)_3_Cl_3_ nanocluster.

Thermogravimetric analysis (TGA) was carried out on a thermogravimetric analyzer (DTG-60H, Shimadzu Instruments, Inc.). 10 mg of clusters was used for collecting the TGA data on clusters.

X-ray photoelectron spectroscopy (XPS) measurements were performed on a Thermo ESCALAB 250 configured with a monochromated Al Kα (1486.8 eV) 150 W X-ray source, 0.5 mm circular spot size, a flood gun to counter charging effects, and analysis chamber base pressure lower than 1 × 10^–9^ mbar.

Inductively coupled plasma-atomic emission spectrometry (ICP-AES) measurements were performed on an Atomscan advantage instrument from Thormo Jarrell Ash Corporation (USA).

Elemental analysis (EA) was performed on Vario EL cube. 3 mg of each cluster sample was used for collecting the EA data.

Energy-dispersive X-ray spectroscopy (EDS) analyses were performed on a JEOL JEM-2100F FEG TEM operated at 200 kV. Nanocluster powder samples were used for the analysis.

Electrospray ionization time-of-flight mass spectrometry (ESI-TOF-MS) measurements were performed using a MicrOTOF-QIII high-resolution mass spectrometer; for preparing the ESI sample, the clusters were dissolved in CH_2_Cl_2_ (1 mg mL^–1^) and diluted (v/v = 1 : 2) with methanol.

## 3 Results and discussion

**Pt_1_Ag_28_** was prepared first *via* an *in situ* synthetic procedure (see the Experimental section for more details). A combination of ESI-MS, UV-vis absorption, PL, XPS, ICP, and EA results unambiguously identified that the obtained **Pt_1_Ag_28_** nanocluster is the same as the one reported previously (Fig. S1, and Tables S1, S2†).8*h* The EDS results demonstrated the presence of Cl in the cluster system, which was considered as the counterion for the **Pt_1_Ag_28_** nanocluster, namely, [**Pt_1_Ag_28_**(S-Adm)_18_(PPh_3_)_4_]Cl_2_ (Fig. S2†).

**Fig. 1:**
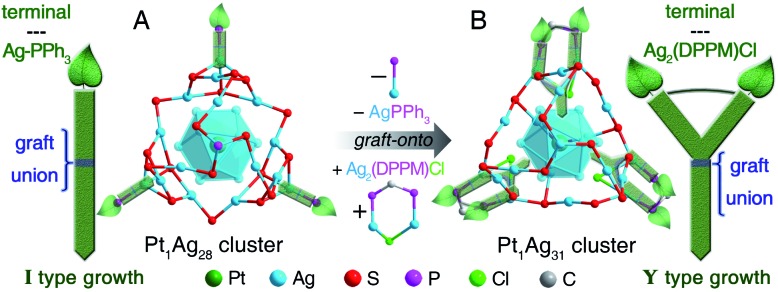
Transformation from **Pt_1_Ag_28_** into **Pt_1_Ag_31_**. (A) Schematic illustration of the I-type growth of the Ag–PPh_3_ terminal and the structure of **Pt_1_Ag_28_**. (B) Schematic illustration of the Y-type growth of the Ag_2_(DPPM)Cl terminal and the structure of **Pt_1_Ag_31_**. In this transformation process, the Ag–PPh_3_ terminals in **Pt_1_Ag_28_** are peeled off, and the Ag_2_(DPPM)Cl terminals are introduced. Color legends: dark green sphere, Pt; blue sphere, Ag; red sphere, S; purple sphere, P; green sphere, Cl; grey sphere, C. For clarity, all H atoms and some C atoms are omitted.

The reaction between **Pt_1_Ag_28_** and Ag_2_(DPPM)Cl_2_ generates a **Pt_1_Ag_31_** nanocluster, wherein the Ag–PPh_3_ vertexes were substituted by the Ag_2_(DPPM)Cl, accompanied by a size-growth of the metallic kernel from M_29_ to M_32_ (M = Pt/Ag; Fig. 1). As for the overall structure, the Ag–PPh_3_ terminals (in **Pt_1_Ag_28_**) are bonded onto the nanoclusters with an I-type growth mode, whereas the Ag_2_(DPPM)Cl terminals (in **Pt_1_Ag_31_**) follow a Y-type growth mode (Fig. 1). The transformation of the nanocluster terminal from the single-linked Ag–PPh_3_ (I type; see Fig. 1A) into the double-linked Ag_2_(DPPM)Cl (Y type; see Fig. 1B) reflects the “graft-onto growth” and accounts for the size growth, structural transformation, and surface rotation of the nanocluster.

The ESI-MS and EDS results identified the molecular formula as [**Pt_1_Ag_31_**(S-Adm)_16_(DPPM)_3_Cl_3_]Cl_4_ (Fig. S3 and S4†). Both **Pt_1_Ag_28_** and **Pt_1_Ag_31_** clusters contain 8 free valence electrons – (i) for **Pt_1_Ag_28_**, the free electron count is 28(Ag) – 18(SR) – 2(charge) = 8; (ii) for **Pt_1_Ag_31_**, the free electron count is 31(Ag) – 16(SR) – 3 (Cl) – 4(charge) = 8. The atomic ratio of Pt to Ag in **Pt_1_Ag_31_** was analyzed by XPS and ICP, and the experimental results were consistent with the theoretical ratio (Fig. S5, and Table S1†). TGA of the **Pt_1_Ag_31_** nanocluster showed a total weight loss of 52.65%, matching the theoretical value of 53.54% (the proportion of SR, DPPM, Cl ligands and the Cl counterion in the overall formula; Fig. S6†).

**Fig. 2:**
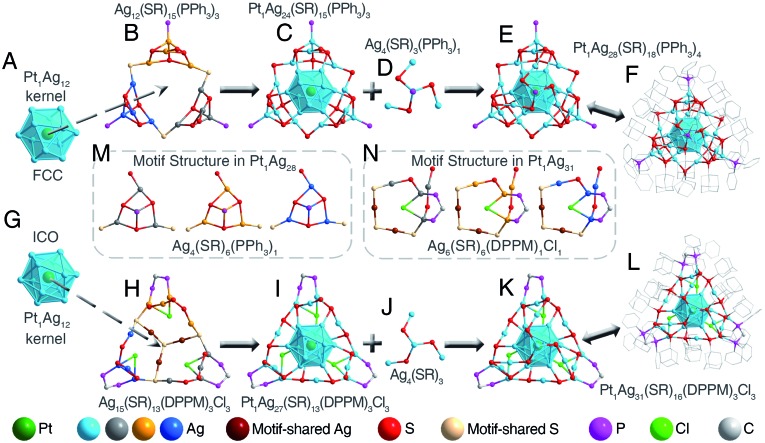
Structural anatomies of **Pt_1_Ag_28_** and **Pt_1_Ag_31_** nanoclusters. (A–F and M) Structural anatomy of the **Pt_1_Ag_28_** nanocluster. (G–L and N) Structural anatomy of the **Pt_1_Ag_31_** nanocluster. Color legends: dark green sphere, Pt; blue/dark grey/orange/dark blue sphere, Ag; brown sphere, motif-shared Ag; red sphere, S; pink sphere, motif-shared S; purple sphere, P; green sphere, Cl; grey sphere, C. For clarity, all H atoms and some C atoms are omitted.

The structural comparison between **Pt_1_Ag_28_** and **Pt_1_Ag_31_** is shown in Fig. 2 (see Fig. S7† for the total structure of **Pt_1_Ag_31_**). **Pt_1_Ag_28_** comprises a fcc Pt_1_Ag_12_ kernel, a trilateral Ag_12_(SR)_15_(PPh_3_)_3_ shell, and a helical Ag_4_(SR)_3_(PPh_3_)_1_ unit (Fig. 2A–F). The trilateral Ag_12_(SR)_15_(PPh_3_)_3_ shell is constituted by assembling of three same Ag_4_(SR)_6_(PPh_3_)_1_ units (Ag_3_(SR)_6_ face + Ag–PPh_3_ terminals) by sharing the terminal thiol ligands (Fig. 2B and M).

With the grafting effect, the vertex Ag-PPh_3_ units on the **Pt_1_Ag_28_** surface are peeled off, and Ag_2_(DPPM)Cl units are introduced. As a result, three additional Ag atoms (**Pt_1_Ag_31_** – **Pt_1_Ag_28_** = 3 Ag) are incorporated onto the nanocluster surface because of the grafting effect of bidentate DPPM ligands. Three Cl ligands are also introduced to further stabilize the surface structure of **Pt_1_Ag_31_**. More specifically—

(i) Although the composition of the Pt_1_Ag_12_ kernel maintains throughout the graft-onto process, the fcc configuration of the Pt_1_Ag_12_ kernel in **Pt_1_Ag_28_** alters to an icosahedral configuration in **Pt_1_Ag_31_** (Fig. 2A and G). The average bond length between Pt(core) and Ag(kernel surface) in **Pt_1_Ag_31_** is smaller than that in **Pt_1_Ag_28_**, whereas the bonds between Ag(kernel surface) and Ag(kernel surface) in **Pt_1_Ag_31_** are much longer than those in **Pt_1_Ag_28_** (Table 1 and Fig. S8†).

**Table 1:** Comparison of bond lengths in **Pt_1_Ag_28_** and **Pt_1_Ag_31_** nanoclusters. Such bonds are highlighted in Fig. S8

	**Pt_1_Ag_28_**	**Pt_1_Ag_31_**	Diff.
Pt(core)–Ag(kernel surface) bond	2.768–2.797 Å (Avg. 2.838 Å)	2.735–2.786 Å (Avg. 2.760 Å)	–2.83%
Ag(kernel surface)–Ag (kernel surface) bond	2.751–2.848 Å (Avg. 2.802 Å)	2.817–3.144 Å (Avg. 2.906 Å)	+3.58%
Ag(kernel surface)–S(motif) bond	2.438–2.498 Å (Avg. 2.472 Å)	2.445–2.591 Å (Avg. 2.495 Å)	+0.92%
Ag(motif)–S(motif) bond	2.254–2.992 Å (Avg. 2.560 Å)	2.356–2.835 Å (Avg. 2.460 Å)	+4.07%
Ag(motif)–P(vertex) bond	2.292–2.384 Å (Avg. 2.356 Å)	2.397–2.428 Å (Avg. 2.405 Å)	+2.04%

(ii) The trilateral Ag_15_(SR)_13_(DPPM)_3_Cl_3_ shell in **Pt_1_Ag_31_** is constituted by the assembly of three same Ag_6_(SR)_6_(DPPM)_1_Cl_1_ units *via* sharing Ag_2_(SR)_3_ edges (Fig. 2H and N). Due to the steric hindrance effect, only 13 thiol ligands exist in the trilateral Ag_15_(SR)_13_(DPPM)_3_Cl_3_ shell and less than the 15 thiol ligands in the Ag_12_(SR)_15_(PPh_3_)_3_ trilateral shell of **Pt_1_Ag_28_** (Fig. 2B and H). In each Ag_6_(SR)_6_(DPPM)_1_Cl_1_ unit, the Cl ligand fixes two Ag atoms that bond with the DPPM ligand (Fig. 2N). All Ag-ligand interactions (including Ag(kernel surface)–S(motif), Ag(motif)–S(motif), and Ag(motif)–P(vertex)) in **Pt_1_Ag_31_** are longer than those in **Pt_1_Ag_28_** (Table 1).

(iii) The Pt_1_Ag_24_(SR)_15_(PPh_3_)_3_ structure in **Pt_1_Ag_28_** is covered by a helical Ag_4_(SR)_3_(PPh_3_)_1_ unit (Fig. 2D), whereas the corresponding structure in **Pt_1_Ag_31_** is just Ag_4_(SR)_3_ (Fig. 2J); that is, the terminal PPh_3_ ligand is peeled off. A similar situation has recently been observed in the transformation of Ag_29_(SSR)_12_(PPh_3_)_4_ into Cs_3_Ag_29_(SSR)_12_(DMF)_*x*_.^11^ For the Cs_3_Ag_29_(SSR)_12_(DMF)_*x*_ nanocluster, because of the absence of the vertex PPh_3_ ligand, the terminal Ag atom becomes closer to the innermost Ag_13_ kernel.^11^ A similar situation has been observed in this work – the average distance between the terminal Ag and adjacent Ag atoms in the innermost Pt_1_Ag_12_ kernel in **Pt_1_Ag_31_** is 4.026 Å, which is much shorter than that in **Pt_1_Ag_28_** (4.290 Å, as shown in Fig. S9†). In this context, the terminal Ag-based structure in **Pt_1_Ag_31_** becomes more contractive for reducing the exposure of this bare Ag atom, which in turn makes the overall structure more robust.

Collectively, the “graft-onto” process on the **Pt_1_Ag_28_** surface changes the vertex structure from PPh_3_-Ag to DPPM-Ag_2_-Cl, resulting in the size-growth and surface structural transformation of the nanocluster. The transformation of the outermost shell further induces changes on kernel–shell interactions, and such changes alter the innermost Pt_1_Ag_12_ kernel from a fcc configuration in **Pt_1_Ag_28_** to an icosahedral configuration in **Pt_1_Ag_31_**.

From the structural point of view, aside from **Pt_1_Ag_28_**(SR)_18_(PPh_3_)_4_, several other metal nanoclusters are terminally capped by metal–PPh_3_, such as Ag_29_(SSR)_12_(PPh_3_)_4_, Ag_33_(SR)_24_(PPh_3_)_4_, Au_23_(PhC≡C)_9_(PPh_3_)_6_, Au_24_(PhC≡C)_14_(PR)_4_, and so on.^8^ Our reported “graft-onto” strategy might also be applicable in these cluster systems for controlling their surface structures. Future work will focus on extending the “graft-onto” strategy to other cluster systems.

Both **Pt_1_Ag_28_** and **Pt_1_Ag_31_** nanoclusters are stable in DMF at 50 °C for at least 24 hours (Fig. S10A and C†). At 80 °C, the optical absorptions of **Pt_1_Ag_28_** disappear over time (Fig. S10B†); by comparison, the **Pt_1_Ag_31_** nanocluster is stable enough to maintain its optical absorptions (Fig. S10D†). We propose that the enhanced thermal stability of **Pt_1_Ag_31_** results from its more robust structure – compared with PPh_3_, the introduced DPPM ligands have more ability to fix the nanocluster surface and thus suppress the vibration of the overall structure.

**Fig. 3:**
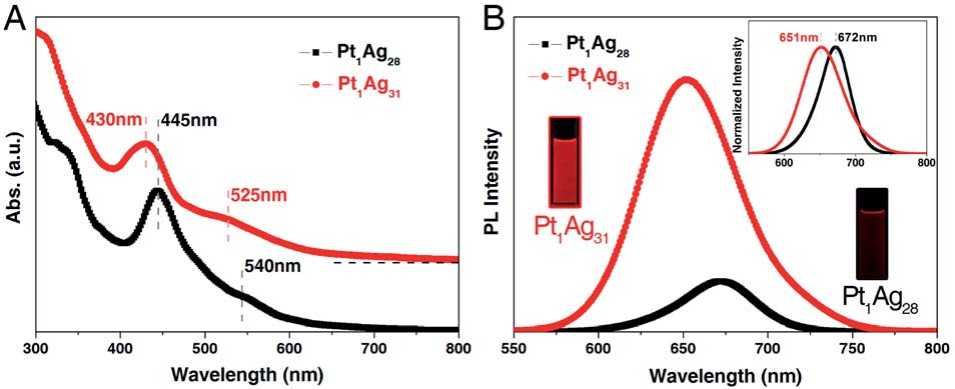
Optical properties of nanoclusters. (A) Optical absorptions of **Pt_1_Ag_28_** and **Pt_1_Ag_31_** nanoclusters. (B) PL emissions of **Pt_1_Ag_28_** and **Pt_1_Ag_31_** nanoclusters. Insets in B: peak shift in normalized PL spectra, and digital photo of each cluster in CH_2_Cl_2_ under UV light.

The optical properties of **Pt_1_Ag_28_** and **Pt_1_Ag_31_** nanoclusters are compared. Optical absorption of **Pt_1_Ag_28_** shows an intense peak at 445 nm and a shoulder peak at 540 nm. The transformation of **Pt_1_Ag_28_** into **Pt_1_Ag_31_** results in an obvious blue-shift for each peak – the peak at 445 nm blue-shifts to 430 nm and becomes wider, and the shoulder band at 540 nm blue-shifts to 525 nm (Fig. 3A). The blue shift of the maximum optical absorption of nanoclusters (*i.e.*, from 540 nm of **Pt_1_Ag_28_** to 525 nm of **Pt_1_Ag_31_**) always represents the enlargement of the HOMO–LUMO energy gap (HOMO: the highest occupied molecular orbital; LUMO: the lowest unoccupied molecular orbital), which matches the DFT calculation results that **Pt_1_Ag_31_** displays a larger energy gap relative to **Pt_1_Ag_28_** (1.92 eV *versus* 1.76 eV, Fig. S11†).

The **Pt_1_Ag_28_** nanocluster (in CH_2_Cl_2_ solution) emits at 672 nm, with a photo-luminescence quantum yield (PL QY) of 4.9%.8*h* By comparison, the **Pt_1_Ag_31_** nanocluster (in CH_2_Cl_2_ solution, with the same OD as **Pt_1_Ag_28_** solution) emits at 651 nm, representing a 21 nm blue shift relative to that of the **Pt_1_Ag_28_**. Significantly, the PL QY of **Pt_1_Ag_31_** in CH_2_Cl_2_ is 29.3%; that is, the PL intensity of **Pt_1_Ag_31_** is six times that of the **Pt_1_Ag_28_**. Such an enhancement can also be inferred from the PL spectra of two nanoclusters (Fig. 3B). Besides, the PL comparison between **Pt_1_Ag_31_** and rhodamine B further determined the PL QY of the nanocluster (Fig. S12 and S13†). Under weak UV light, the emission of **Pt_1_Ag_28_** is hard to be observed, whereas the PL of **Pt_1_Ag_31_** is strong enough to be perceived with the naked eye (Fig. 3B, insets). Such a PL enhancement may result from the enhanced stabilization ability of DPPM relative to PPh_3_ – the introduced DPPM ligand firmly fixes the surface structure and suppresses the vibration of the overall structure; in this context, the energy dissipation of the photo-excited **Pt_1_Ag_31_** reduces with non-radiative transitions (mainly affected by intramolecular vibrations), but enhances with radiative transitions (through luminescence).

**Fig. 4:**
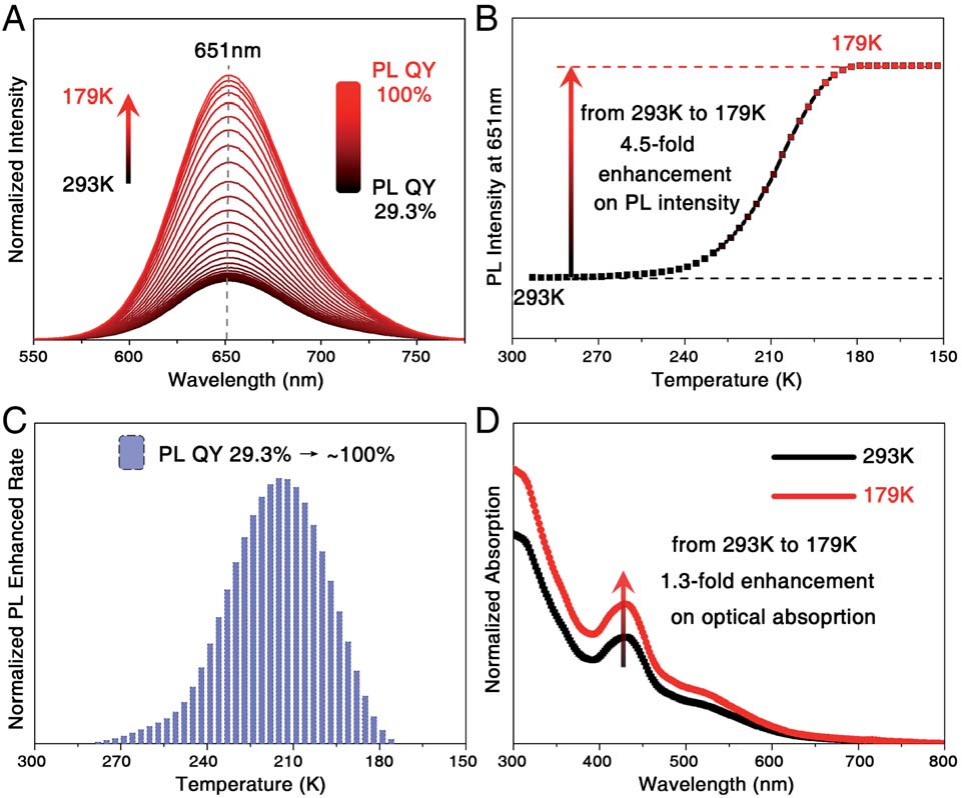
Temperature-dependent PL of **Pt_1_Ag_31_** (dissolved in CH_2_Cl_2_). (A) Temperature-dependent emission of **Pt_1_Ag_31_**. (B) The PL intensity of **Pt_1_Ag_31_** at the fixed point of 651 nm. (C) The derivative results for the temperature-dependent PL intensity of **Pt_1_Ag_31_**. (D) Optical absorption of **Pt_1_Ag_31_** at 293 K and 179 K.

Temperature-dependent fluorescence of the **Pt_1_Ag_31_** nanocluster was monitored. For the **Pt_1_Ag_28_** nanocluster, our previous work demonstrated a 20-fold enhancement on the PL intensity of **Pt_1_Ag_28_** (in DMF solution) along with the temperature-lowering process from 293 K to 125 K, and the PL QY increased from 9.3% to ∼100% (Fig. S14†).12*a* As to the **Pt_1_Ag_31_** nanocluster (Fig. 4), the PL intensity presented a 4.5-fold enhancement by comparing the 179 K data with the 293 K data (Fig. 4A–C), and the optical absorption just exhibited a 1.3-fold enhancement in the corresponding temperature-lowering process (Fig. 4D). Accordingly, the PL QY of **Pt_1_Ag_31_** was almost 100% when the temperature was lower than 179 K. Such an enhancement of PL intensity resulted from the reduced energy consumption of thermal vibrations of nanoclusters (non-radiative transition) reduced by the reduced temperature; in this context, the excitation energy could only be released by the PL approach (radiative transition).^11^,^12^

## 4 Conclusions

In summary, a “graft-onto” strategy is presented for facilitating a controllable size-growth of the nanocluster surface. The addition of the Ag_2_(DPPM)Cl_2_ complex converts **Pt_1_Ag_28_**(S-Adm)_18_(PPh_3_)_4_ into a size-growth nanocluster, namely, **Pt_1_Ag_31_**(S-Adm)_16_(DPPM)_3_Cl_3_. Induced by the grafting effect, direct changes on the surface structure (*e.g.*, size growth, structural transformation, and surface rotation) and indirect changes on the kernel structure (from a fcc configuration to an icosahedral configuration) take place. Obvious differences have been observed by comparing the optical properties (optical absorption and PL emission) of two nanoclusters. Significantly, **Pt_1_Ag_31_**(SR)_16_(DPPM)_3_Cl_3_ displayed a high PL intensity with a PL QY of 29.3%, which is six times that of the **Pt_1_Ag_28_**(SR)_18_(PPh_3_)_4_. Our work presents a new strategy for controllably re-constructing the nanocluster surface at the atomic level, which hopefully sheds light on the fundamental understanding of how surface structures in a nanocluster contribute to its overall properties.

## Conflicts of interest

There are no conflicts to declare.

## Supplementary Material

Supplementary information

Crystal structure data
