# Supplementary material for: Nanocluster growth via “graft-onto”: effects on geometric structures and optical properties
Source: Chem Sci. 2019 Dec 27;11(6):1691–7. doi: 10.1039/c9sc05700e (PMC7069245; doi:10.1039/c9sc05700e)
Supplement: Supplementary file 1 [file SC-011-C9SC05700E-s001.pdf]

Electronic Supplementary Material (ESI) for Chemical Science.
This journal is © The Royal Society of Chemistry 2019

Supporting Information

Nanocluster growth *via* "graft-onto": effects on geometric structures and optical properties

Xi Kang,[‡,a,c] Shan Jin,[‡,b] Lin Xiong,[d] Xiao Wei,[a,c] Manman Zhou,[a,c] Chenwanli Qin,[a,c] Yong Pei,[d] Shuxin Wang,*[,a,c] Manzhou Zhu*[,a,b,c]

[a]Department of Chemistry and Centre for Atomic Engineering of Advanced Materials, Anhui Province Key Laboratory of Chemistry for Inorganic/Organic Hybrid Functionalized Materials, Anhui University, Hefei, Anhui, 230601, P. R. China.
[b]Institutes of Physical Science and Information Technology, Anhui University, Hefei, Anhui 230601, P. R. China.
[c]Key Laboratory of Structure and Functional Regulation of Hybrid Materials, Anhui University, Ministry of Education, Hefei, 230601, P. R. China.
[d]Department of Chemistry, Key Laboratory of Environmentally Friendly Chemistry and Applications of Ministry of Education, Xiangtan University, Xiangtan, Hunan 411105, China.

[‡]X.K. and S.J. contributed equally.

*E-mails of corresponding authors: ixing@ahu.edu.cn (S.W.); zmz@ahu.edu.cn (M.Z.).

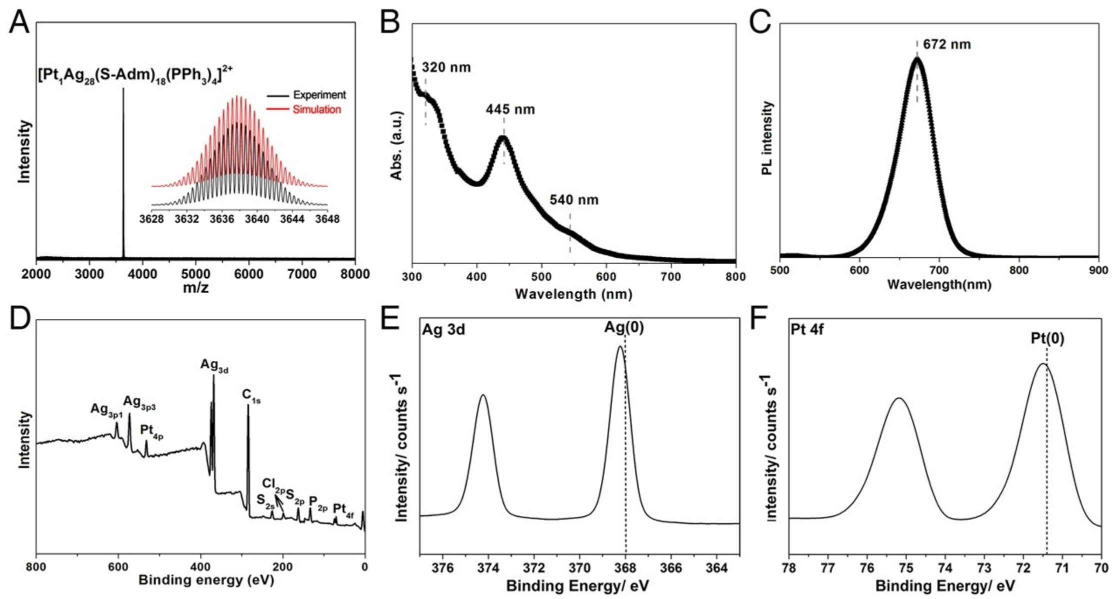

Fig. S1 Characterizations of the $[Pt_1Ag_{28}(SR)_{18}(PPh_3)_4]Cl_2$ nanocluster. (A) ESI-MS. (B) UV-vis absorption. (C) PL emission. (D-F) XPS.

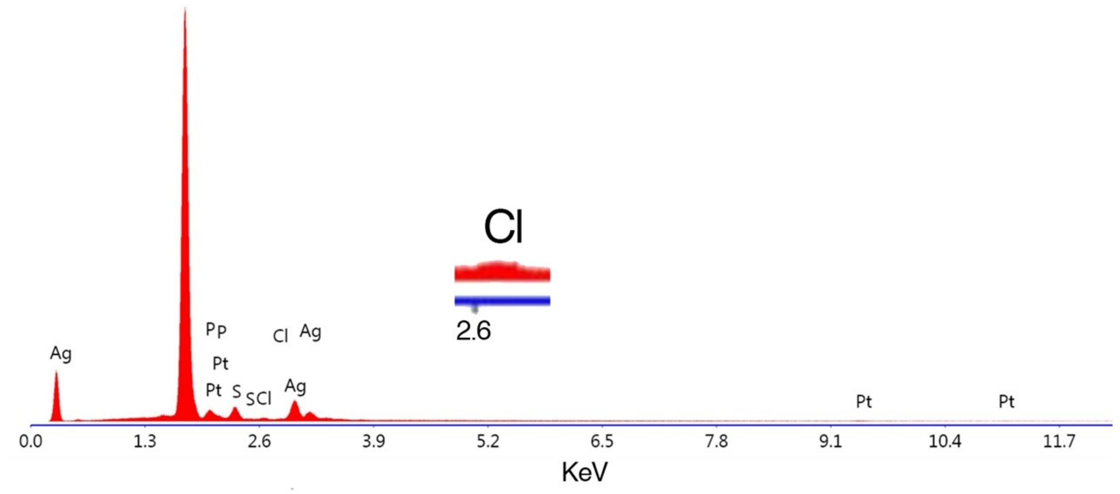

Fig. S2 EDS result of the $[Pt_1Ag_{28}(SR)_{18}(PPh_3)_4]Cl_2$ nanocluster. The Cl peak is weak in the spectrum, because of the low proportion of Cl atom in $[Pt_1Ag_{28}(SR)_{18}(PPh_3)_4]Cl_2$.

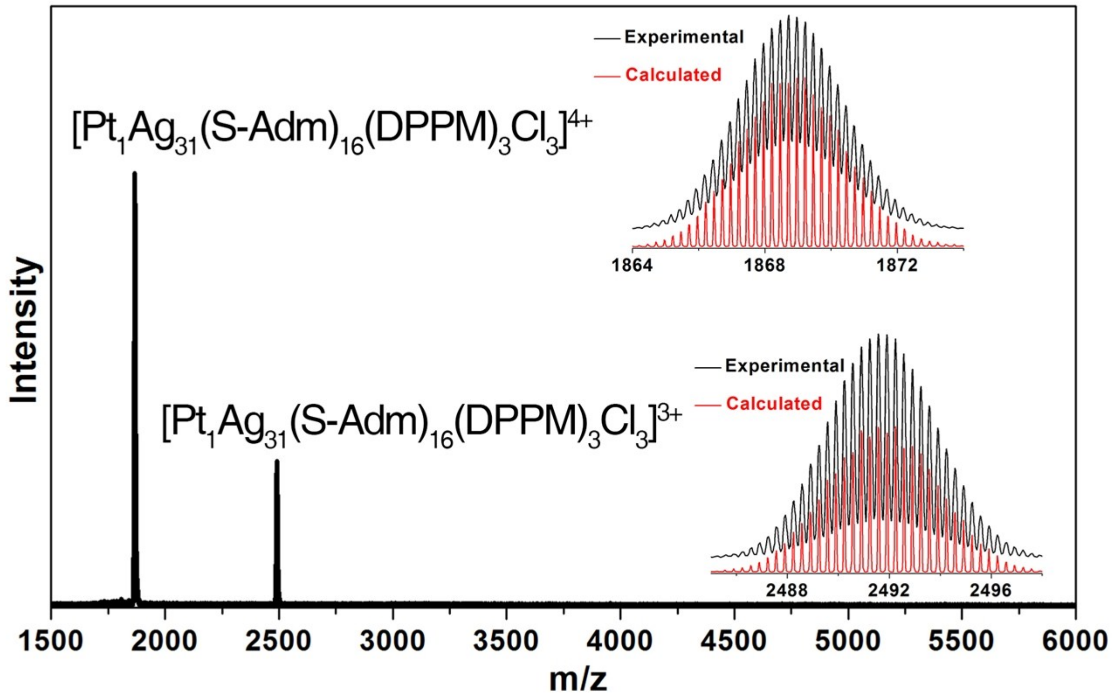

Fig. S3 ESI-MS result of the $[Pt_1Ag_{31}(SR)_{16}(DPPM)_3Cl_3]Cl_4$ nanocluster. Insets: experimental and simulated mass spectra of $[Pt_1Ag_{31}(SR)_{16}(DPPM)_3Cl_3]^{4+}$ and $[Pt_1Ag_{31}(SR)_{16}(DPPM)_3Cl_3]^{3+}$.

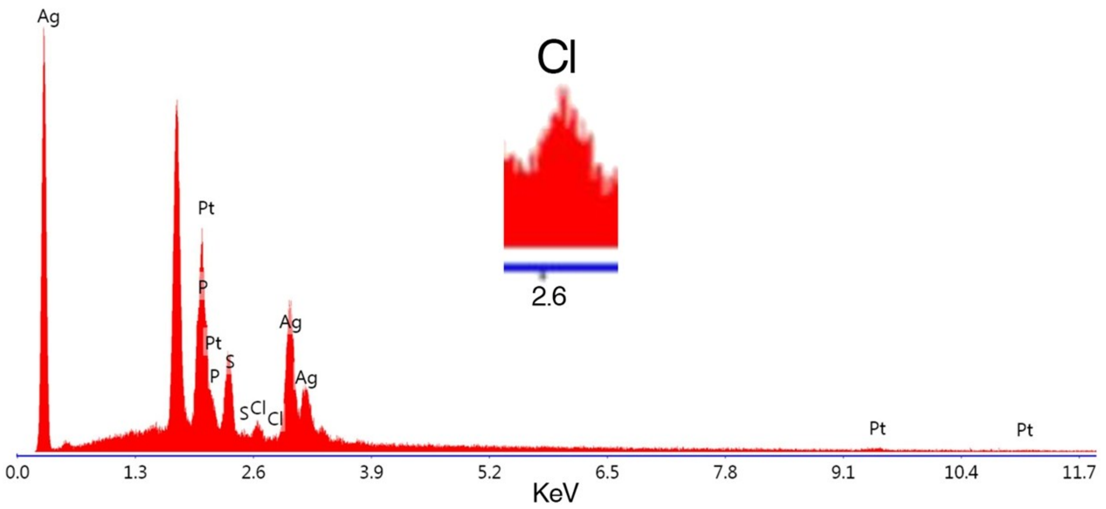

Fig. S4 EDS result of the $[Pt_1Ag_{31}(SR)_{16}(DPPM)_3Cl_3]Cl_4$ nanocluster. The Cl signal in the EDS spectrum of $[Pt_1Ag_{31}(SR)_{16}(DPPM)_3Cl_3]Cl_4$ is much stronger than that in the spectrum of $[Pt_1Ag_{28}(SR)_{18}(PPh_3)_4]Cl_2$, because the number Cl atoms in $[Pt_1Ag_{31}(SR)_{16}(DPPM)_3Cl_3]Cl_4$ is 3.5 times that of the $[Pt_1Ag_{28}(SR)_{18}(PPh_3)_4]Cl_2$ nanocluster.

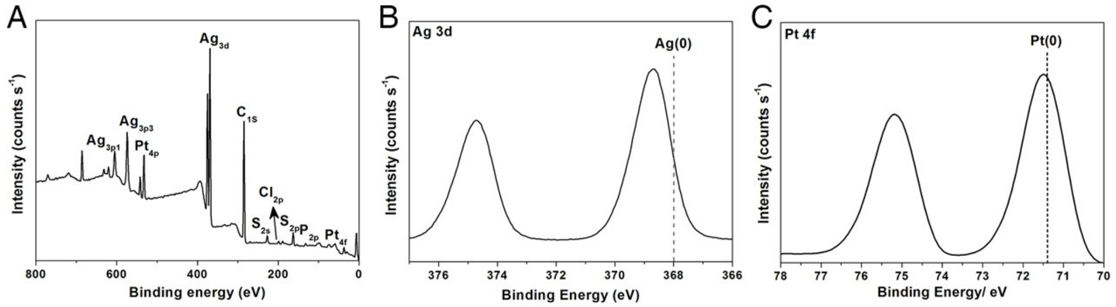

Fig. S5 XPS results of the $[Pt_1Ag_{31}(SR)_{16}(DPPM)_3Cl_3]Cl_4$ nanocluster.

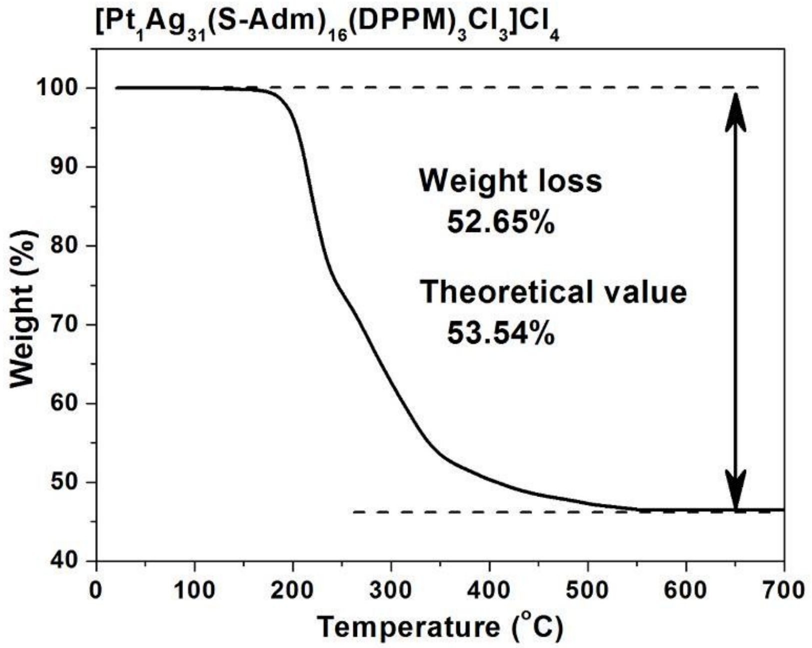

Fig. S6 TGA result of the $[Pt_1Ag_{31}(SR)_{16}(DPPM)_3Cl_3]Cl_4$ nanocluster. The weight loss is 52.65%, matching with the theoretical value of 53.54%.

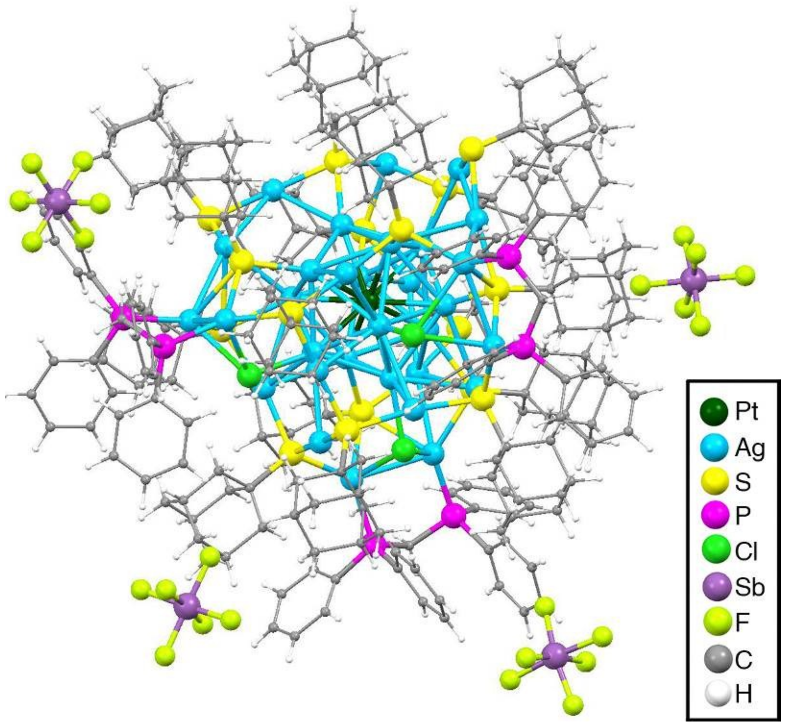

Fig. S7 Total structure of the $[Pt_1Ag_{31}(S\text{-}Adm)_{16}(DPPM)_3Cl_3](SbF_6)_4$ nanocluster.

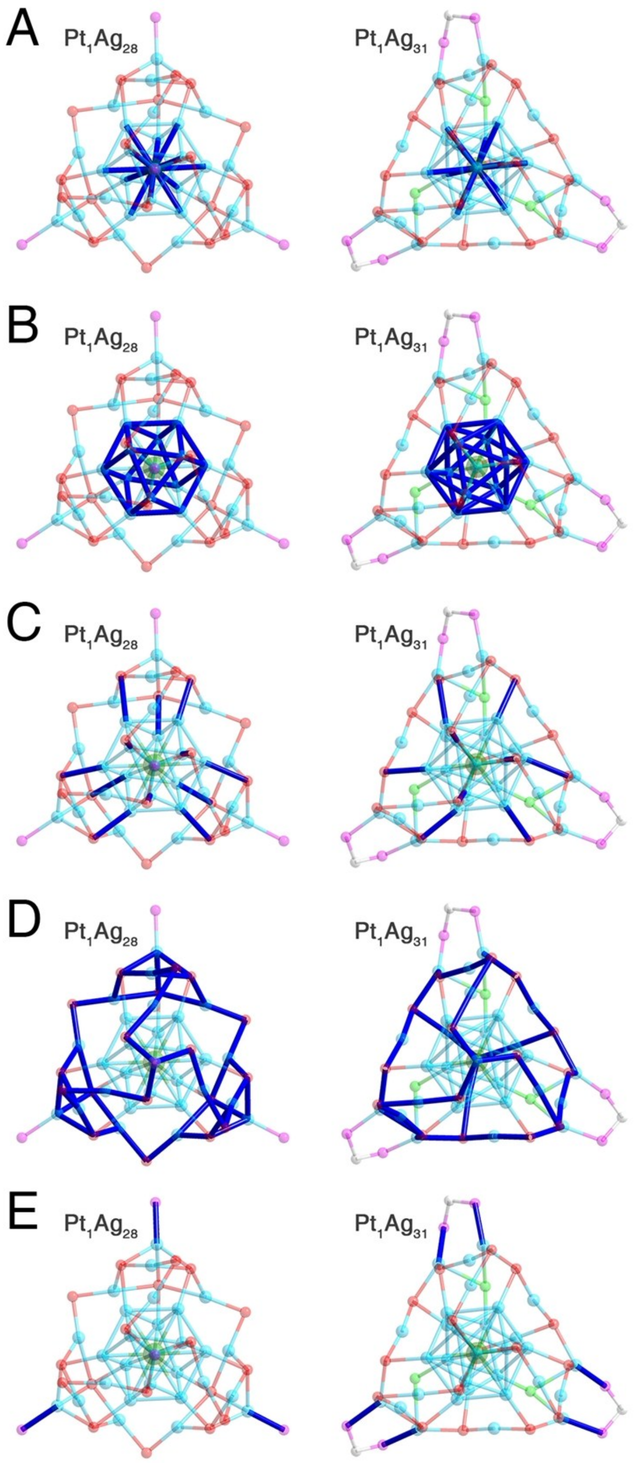

Fig. S8 (A) Bonds between Pt(core) and Ag(kernel surface) (highlighted in dark blue). (B) Bonds between Ag(kernel surface) and Ag (kernel surface) (highlighted in dark blue). (C) Bonds between Ag(kernel surface) and S(motif) (highlighted in dark blue). (D) Bonds between Ag(motif) and S(motif) (highlighted in dark blue). (E) Bonds between Ag(motif) and P (highlighted in dark blue).

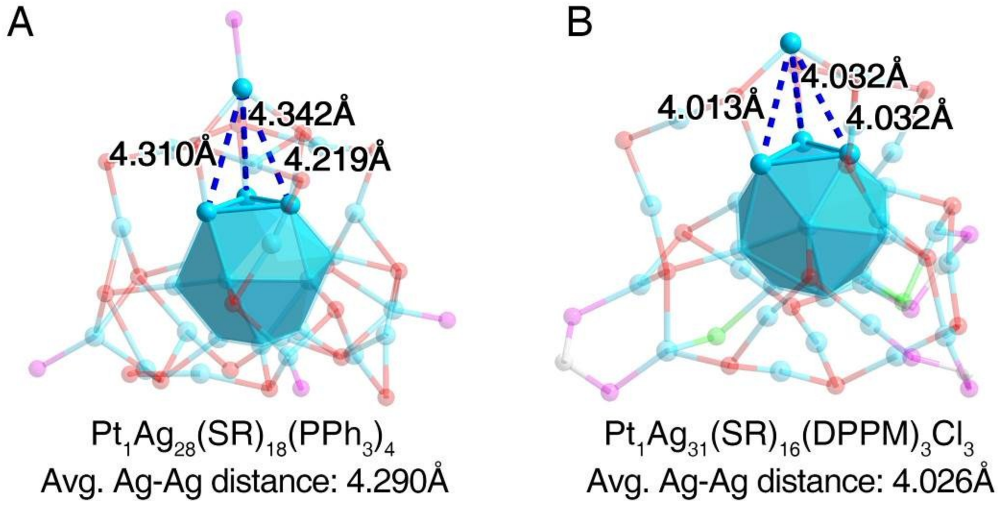

Fig. S9 Comparison of the bond length between the vertex Ag and the icosahedral surface Ag in (A) $Pt_1Ag_{28}(SR)_{18}(PPh_3)_4$ and (B) $Pt_1Ag_{31}(SR)_{16}(DPPM)_3Cl_3$ nanoclusters.

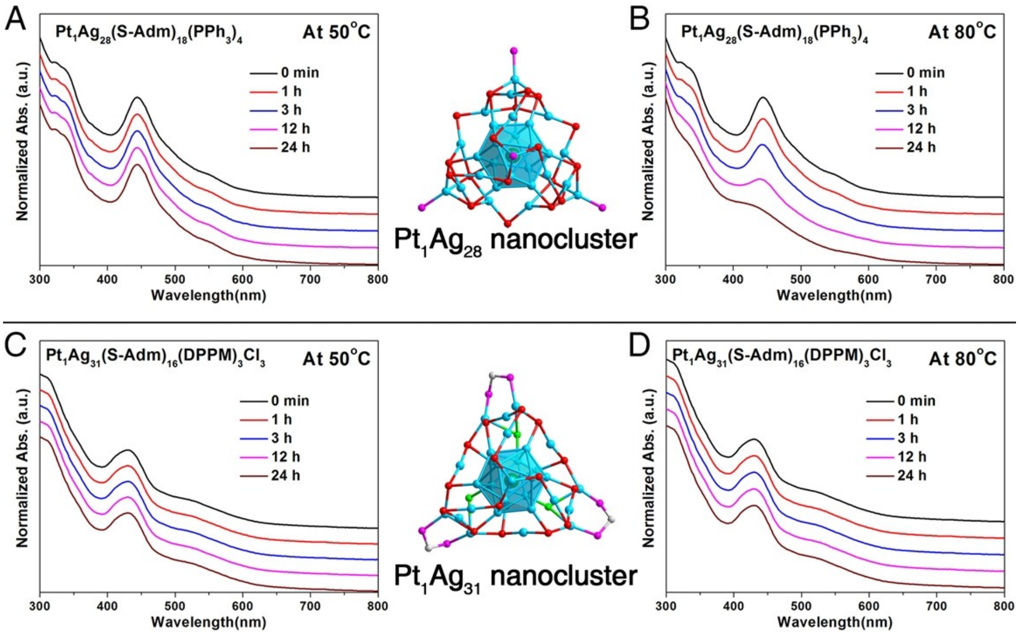

Fig. S10 Thermal stability of the $Pt_1Ag_{28}(SR)_{18}(PPh_3)_4$ nanocluster at (A) 50^oC and (B) 80^oC. Thermal stability of the $Pt_1Ag_{31}(SR)_{16}(DPPM)_3Cl_3$ nanocluster at (C) 50^oC and (D) 80^oC.

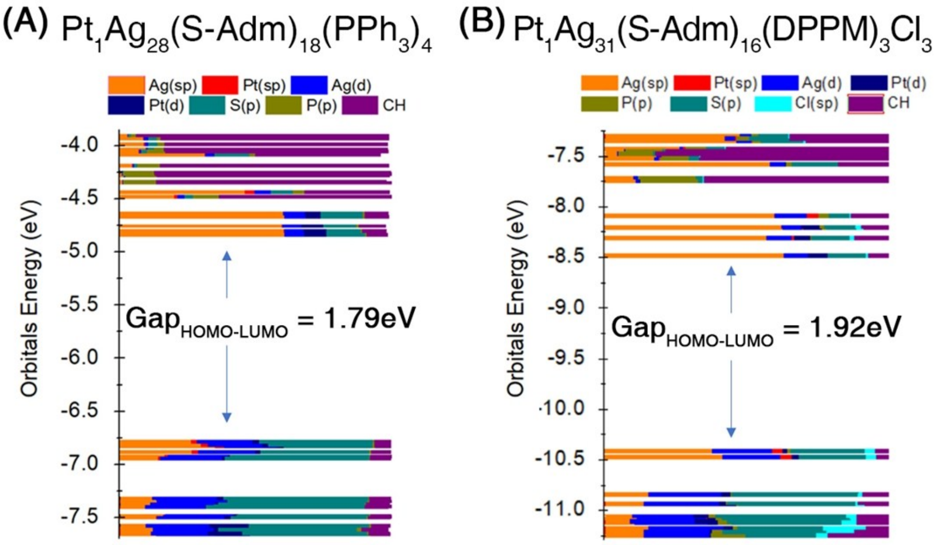

Fig. S11 Kohn-Sham molecular orbitals (MO) of (A) $Pt_1Ag_{28}(SR)_{18}(PPh_3)_4$ and (B) $Pt_1Ag_{31}(SR)_{16}(DPPM)_3Cl_3$. The HOMO-LUMO energy gap of Pt_1Ag_{28} is 1.79 eV, lower than that of Pt_1Ag_{31} (1.92 eV).

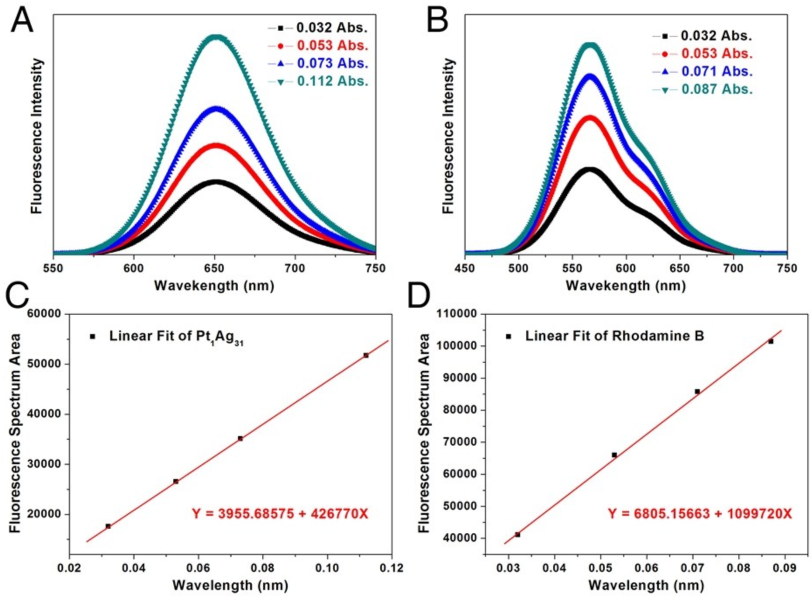

Fig. S12 (A) Fluorescence spectra of the $Pt_1Ag_{31}(SR)_{16}(DPPM)_3Cl_3$ nanocluster with different absorbance (in CH_2Cl_2 solution). (B) Fluorescence spectra of Rhodamine B with different absorbance (in CH_3CH_2OH solution). (C) Absorbance vs. area of fluorescence of the $Pt_1Ag_{31}(SR)_{16}(DPPM)_3Cl_3$ nanocluster. (D) Absorbance vs. area of fluorescence of Rhodamine B. The quantum yield (QY) of the Pt_1Ag_{31} nanocluster can be calculated using the formula of: $QY(Pt_1Ag_{31}) = QY(Rhodamine\ B) * (Grad_{cluster}/Grad_{Rhodamine\ B}) * [(n_{CH2Cl2})^2/(n_{CH3CH2OH})^2]$, wherein QY(Rhodamine B) is 69%; $Grad_{cluster}$ and $Grad_{Rhodamine\ B}$ are 426770 and 1099720, respectively, according to Fig. S10C,D; n is the refractive index of the solvent, and n_{CH2Cl2} and $n_{CH3CH2OH}$ are 1.4241 and 1.3614, respectively. In this context, the PL QY of Pt_1Ag_{31} nanocluster is 29.3%.

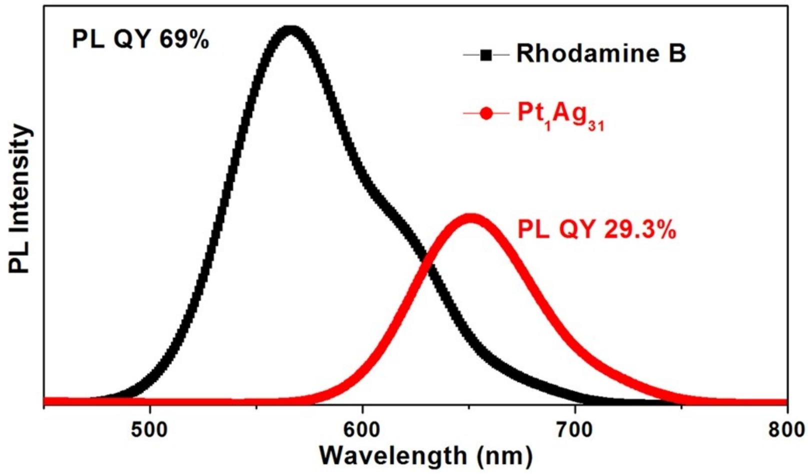

Fig. S13 Comparison of the PL spectra between $Pt_1Ag_{31}(SR)_{16}(DPPM)_3Cl_3$ nanocluster (in CH_2Cl_2) and Rhodamine B (in CH_3CH_2OH) with the same absorption (0.053 Abs.) at the emissive point.

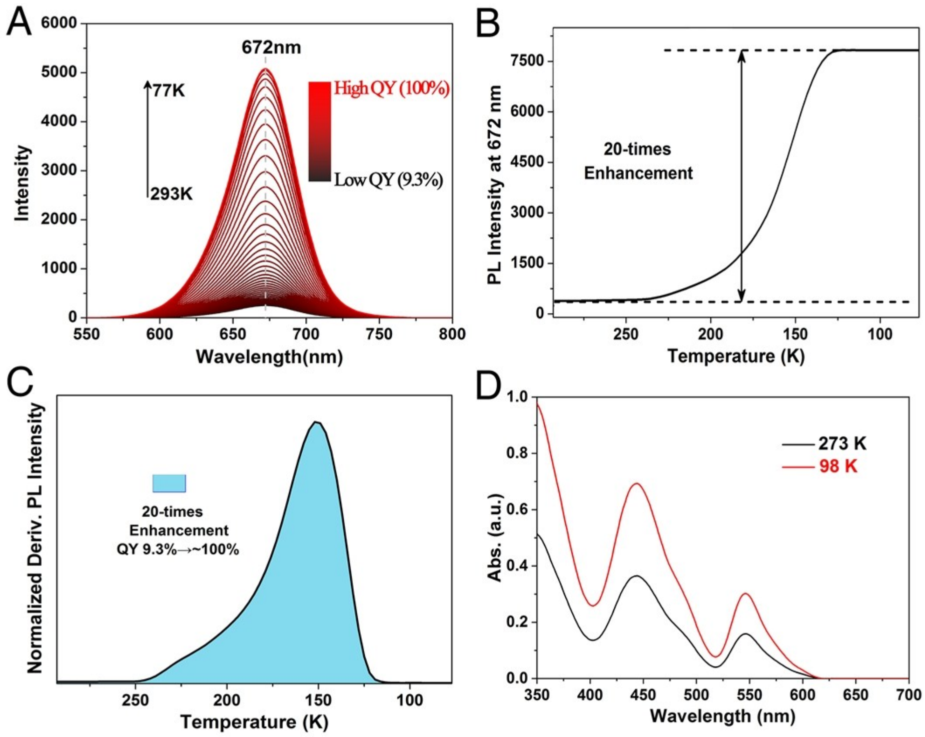

Fig. S14 (A) PL variation of the $Pt_1Ag_{28}(S\text{-}Adm)_{18}(PPh_3)_4$ nanocluster accompanying by the reduction of the temperature (from 293 K to 77 K, monitored per 3 K). (B) PL intensity on the fixed point of 672 nm of the $Pt_1Ag_{28}(S\text{-}Adm)_{18}(PPh_3)_4$ nanocluster under different temperatures. (C) Derivative result on the PL intensity of the $Pt_1Ag_{28}(S\text{-}Adm)_{18}(PPh_3)_4$ nanocluster. (D) Temperature-dependent UV-vis absorptions of the $Pt_1Ag_{28}(S\text{-}Adm)_{18}(PPh_3)_4$ nanocluster.

Table S1. Atom ratio of Pt and Ag in Pt_1Ag_{28} and Pt_1Ag_{31} nanoclusters. Atom ratios of Pt and Ag in $Pt_1Ag_{28}(S\text{-}Adm)_{18}(PPh_3)_4$ and $Pt_1Ag_{31}(S\text{-}Adm)_{16}(DPPM)_3Cl_3$ nanoclusters were calculated from inductively coupled plasma (ICP) and X-ray photoelectric spectroscopy (XPS).

$Pt_1Ag_{28}(S\text{-}Adm)_{18}(PPh_3)_4$	Pt atom	Ag atom
ICP Experiment Ratio	3.90%	96.10%
XPS Experiment Ratio	3.35%	96.65%
Theoretical Ratio	3.45%	96.55%

$Pt_1Ag_{31}(S\text{-}Adm)_{16}(DPPM)_3Cl_3$	Pt atom	Ag atom
ICP Experiment Ratio	3.25%	96.65%
XPS Experiment Ratio	3.17%	96.83%
Theoretical Ratio	3.13%	96.87%

Table S2. Elemental C and H contents in Pt_1Ag_{28} and Pt_1Ag_{31} nanoclusters. Elemental C and H contents in $Pt_1Ag_{28}(S\text{-}Adm)_{18}(PPh_3)_4$ and $Pt_1Ag_{31}(S\text{-}Adm)_{16}(DPPM)_3Cl_3$ nanoclusters were measured by elemental analysis (EA).

$[Pt_1Ag_{28}(S\text{-}Adm)_{18}(PPh_3)_4]Cl_2$	C wt%	H wt%
Experiment Result	40.11%	4.49%
Theoretical Result	41.19%	4.49%

$[Pt_1Ag_{31}(S\text{-}Adm)_{16}(DPPM)_3Cl_3]Cl_4$	C wt%	H wt%
Experiment Result	36.16%	3.87%
Theoretical Result	37.05%	4.02%

Table S3. Crystal data and structure refinement for the Pt_1Ag_{31}(S-Adm)$_{16}$(DPPM)$_3$$Cl_3$ nanocluster.

Crystal system	triclinic
Space group	P -1
a/Å	21.5150(17)
b/Å	24.1156(17)
c/Å	35.321(3)
α/°	81.037(4)
β/°	78.006(4)
γ/°	68.595(4)
Volume/Å^3	16625(2)
Z	2
ρ_{calc}g/cm^3	1.728
μ/mm^{-1}	2.737
F(000)	8386.0
Crystal size/mm^3	0.1 × 0.1 × 0.1
Radiation	MoKα (λ = 0.71073)
Index ranges	-24 ≤ h ≤ 25, -28 ≤ k ≤ 28, -36 ≤ l ≤ 41
Final R indexes [I>=2σ (I)]	R_1 = 0.0746, wR_2 = 0.1872
Final R indexes [all data]	R_1 = 0.1297, wR_2 = 0.2093
